# Design optimization of transmitting antennas for weakly coupled magnetic induction communication systems

**DOI:** 10.1371/journal.pone.0171982

**Published:** 2017-02-13

**Authors:** Nikolay Tal, Yahav Morag, Lisa Shatz, Yoash Levron

**Affiliations:** 1 Department of Electrical Engineering, Technion—Institute of Technology, Haifa, Israel; 2 Department of Engineering, Suffolk University, Boston, Massachusetts, United States of America; Nankai University, CHINA

## Abstract

This work focuses on the design of transmitting coils in weakly coupled magnetic induction communication systems. We propose several optimization methods that reduce the active, reactive and apparent power consumption of the coil. These problems are formulated as minimization problems, in which the power consumed by the transmitting coil is minimized, under the constraint of providing a required magnetic field at the receiver location. We develop efficient numeric and analytic methods to solve the resulting problems, which are of high dimension, and in certain cases non-convex. For the objective of minimal reactive power an analytic solution for the optimal current distribution in flat disc transmitting coils is provided. This problem is extended to general three-dimensional coils, for which we develop an expression for the optimal current distribution. Considering the objective of minimal apparent power, a method is developed to reduce the computational complexity of the problem by transforming it to an equivalent problem of lower dimension, allowing a quick and accurate numeric solution. These results are verified experimentally by testing a number of coil geometries. The results obtained allow reduced power consumption and increased performances in magnetic induction communication systems. Specifically, for wideband systems, an optimal design of the transmitter coil reduces the peak instantaneous power provided by the transmitter circuitry, and thus reduces its size, complexity and cost.

## Introduction

Magnetic induction (MI) communication systems use magnetic coupling to transmit data between two compact, non-radiative coils. The magnetic field produced by the coils decays in proportion to 1/*r*^3^ [1], [2], thus allowing secured communication ‘bubbles’ and frequency reuse [1]. Magnetic communications offer significant advantages in short range applications. One advantage is low power consumption, since the coils employed at MI systems are poor radiators, and communication is achieved by the non-radiative near field. Hence, the efficiency of such systems depends only on the losses in the transmitting coil, and the electronic circuitry driving it. Additional advantages of magnetic induction communications are the elimination of multipath fading and shadowing [3–6]. In addition, since quasi-static magnetic fields penetrate conductive substances, they may be used to communicate through conductive materials such as water [5], the human body (BAN) [7–10], or the earth [11–14].

Two main challenges associated with the design of magnetic induction communication systems are range extension, and channel capacity maximization. Methods to address these challenges include: (a) optimal coils design, (b) increasing the sensitivity of the receiver, and (c) magnetic waveguides. The first two methods are appropriate for MI communication systems operating across both constant and dynamically changing channels. The third method is appropriate for a constant channel, such as in mines or buried sensors communication, and for multiple access applications.

The structure of the transceiver coils has a significant effect on system performance [15] [16]. Work [15] investigates the impact of the antenna topology on the communication link, and analyzes the effects of different parameters such as coil radius, quality factor, and permeability. It is shown in [15] and [16] that the increase in quality factor does not increase the capacity, due to a non-linear decrease in bandwidth. In [17] an improvement of the transponder antenna matching is investigated, and numerical analysis is proposed to compute the magnetic parameters of multi-antenna systems.

One method to extend the range of MI communication employs relaying coils, commonly referred as magnetic waveguides [5], [10–14], [18], [19]. These relays are deployed along the communication channel and enable the extension of the range of magnetic transmission through soil or water. A magnetic channel for underwater communication is modeled in [5], and a waveguide technique is proposed to reduce path losses. Concerning body area network applications, it is shown in [10] that MI relay networks overcome the problem of dead spots, and extend the range without increasing the transmission power. For underground environments, a suitable channel model and waveguide are developed in [11–14]. These works consider optimal deployment of the waveguide relay coils and MI network system parameters, with an attempt to maximize the system throughput. MI waveguide channels are investigated in [18], with a focus on multipath caused by far and near relays coupling to the receiver. It is shown in [19] that magnetic induction networks consisting of multiple coils allow increased range and robustness against coil orientation mismatch in multiple access configurations.

This work proposes a method to increase the efficiency of MI communication transmitting coils, for applications in which the receiver and transmitter are weakly coupled, such as BAN, underground and underwater applications. We develop several optimization methods that reduce the active power losses in the transmitter, along with its reactive power consumption. These problems are formulated as minimization problems, in which the active, reactive or apparent power consumed by the transmitting coil are minimized, under the constraint of providing a required magnetic field at the receiver location. The decision variables are the electric current densities in the volume of the transmitting coil. Since the resulting problems are of high dimension, and in certain cases non-convex, the purpose of this work is to provide efficient numeric and analytic solution methods.

The first problem considered is minimization of reactive power in a flat disc transmitter coil. This problem is solved by the Lagrange multipliers method, which provides an analytic solution for the optimal current distribution across the disc. This example is then extended to general three dimensional transmitter coils with arbitrary topologies. The two methods are shown to provide similar results for flat disc coils. These results extend previous works presented in [20], [21]. Finally, the problem of minimizing the total apparent power consumed by the transmitter coil is addressed. We develop a method to reduce the computational complexity of this problem by transforming it to an equivalent problem of lower dimension, allowing a quick and accurate numeric solution. The results obtained allow reduced power consumption and increased performances in magnetic induction communication systems. Specifically, for wideband systems, an optimal design of the transmitter coil reduces the peak instantaneous power provided by the transmitter circuitry, and thus reduces its size, complexity and cost. It is also shown that for resonant narrowband systems, the proposed method reduces the inductance of the transmitting coil, thus decreasing the quality factor of the resonating LC network, and hence increasing the bandwidth and channel capacity.

## Reactive power in magnetic communication systems

At certain applications, especially when power or data are transmitted over long ranges, the reactive power of the magnetic transmitter may be considerably higher than its active power. When the magnetic transmitter and receiver are far apart, only small part of the magnetic field links the transmitting and receiving coils. As a result, the reactive power, which is proportional to the rate of change in the magnetic energy stored in the medium, may be higher than the active power delivered to the receiving coil. When reactive power is dominant, it may determine the physical size of the transmitter and its cost. The reactive power determines voltages and currents magnitudes and thus affects the conduction and switching losses in the corresponding electronic circuitry and coil. For the same reasons, the reactive power also determines the size and cost of capacitors used for reactive power compensation.

**Fig 1:**
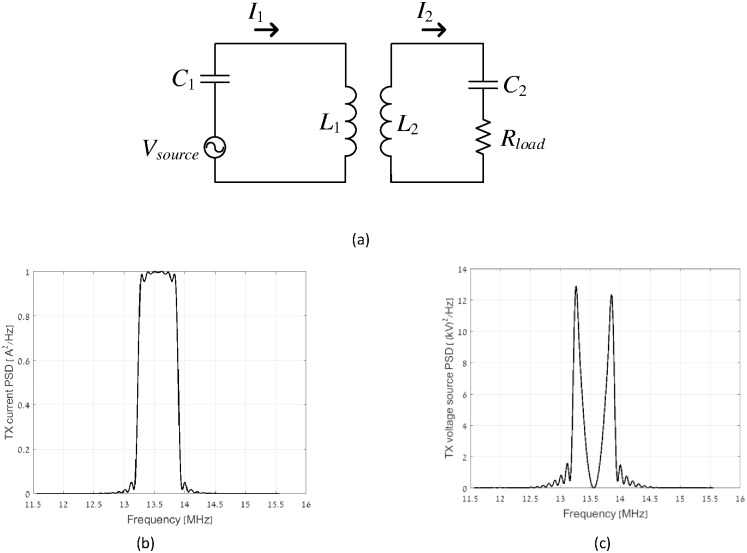
An Example showing the effect of resonance capacitors in broadband magnetic communication. (a) Circuit schematic, (b) Current PSD, and (c) Corresponding voltage PSD resonant communication system.

In power transfer applications, which typically operate at a single frequency, reactive power may be effectively eliminated by means of resonance capacitors. However, in communication systems, this approach may limit the useful bandwidth, since the quality factor of the resonance circuit is proportional to the square root of inductance over the resonating capacitance. Consider for example a weakly coupled Near Field Communication (NFC) system operating at center frequency of 13.56 MHz, with 10 carriers and symbol duration of 10 μs, schematically depicted in Fig 1(a).

The power spectral density (PSD) of the transmitter current *I*_1_ is shown in Fig 1(b) and the resultant PSD of the voltage source *V*_*source*_ is shown in Fig 1(c). It can be inferred observing Fig 1(c) that the resonant capacitor *C*_1_ is not able to compensate for the reactive power at the desired wideband spectrum, resulting in large voltage magnitudes at the source.

The following analysis shows that minimizing the ratio of reactive power to magnetic field at given distance is proportional to minimization of inductance over effective area. To show this, assume an axial magnetic field of a single turn loop. The magnetic field is expressed in terms of magnetic moment [2] as

$$H_{z}=\frac{m}{2\pi z^{3}}$$ (1)

Where z is the axial distance and the magnetic moment *m* is defined as

$$m=\left(\text{loop area}\right)\cdot I_{0}$$ (2)

and *I*_0_ is the current magnitude. The total axial magnetic field resulting from *N* turns is

$$H_{Z,T}=\frac{\Sigma_{i=1}^{N}m_{i}}{2\pi z^{3}}=\frac{m_{total}}{2\pi z^{3}}$$ (3)

Where *m*_total_ is the total magnetic moment of the coil and *H*_*Z*,*T*_ is the desired magnetic field at a certain distance *z*. The transmitter effective area is defined using the total magnetic moment by

$$A_{eff}=\frac{\Sigma_{i=1}^{N}m}{I_{0}}$$ (4)

The reactive power of the coil is given by

$$Q=\frac{1}{2}\omega LI_{0}{}^{2}$$ (5)

Where *ω* is the angular frequency and *L* is the coil inductance. Eqs (3)–(5) lead to the ratio:

$$\frac{\sqrt{Q}}{H_{Z,T}}=\pi z^{3}\sqrt{2\omega }\frac{\sqrt{L}}{\frac{\Sigma_{i=1}^{N}m}{I_{0}}}=\pi z^{3}\sqrt{2\omega }\frac{\sqrt{L}}{A_{eff}}$$ (6)

Hence, reduction of reactive power per desired magnetic field is equivalent to reduction of inductance per effective area. As a result, by reducing the inductance per effective area the quality factor decreases, and the communication bandwidth increases.

## Minimal reactive power in a flat disc transmitting coil

This section focuses on obtaining minimal reactive power in a flat disc topology, which is of practical significance in volume limited applications. This problem is solved analytically by Lagrange multipliers, demonstrating the proposed approach. The problem is formulated as a constrained optimization problem, whose objective is to minimize the magnetic energy, under the constraint of a certain magnetic field at the receiver location. The general problem is formulated by

$$\begin{array}{l} \text{min}.\;W=\frac{\mu_{0}}{4}{\underset{\begin{array}{l} \text{ all}\\ \text{space} \end{array}}{∭}\left|\vec{H}(\vec{p})\right|}^{2}dv\\ \text{s.t. }\left|H_{z}(z=z_{\text{receiver}})\right|=H_{z}{}^{rec} \end{array}$$ (7)

Where *H* is the magnetic field, *H*_*z*_^*rec*^ is the magnetic field magnitude at the receiver location on the z axis, and *W* is the average energy, which is proportional to the reactive power.

**Fig 2:**
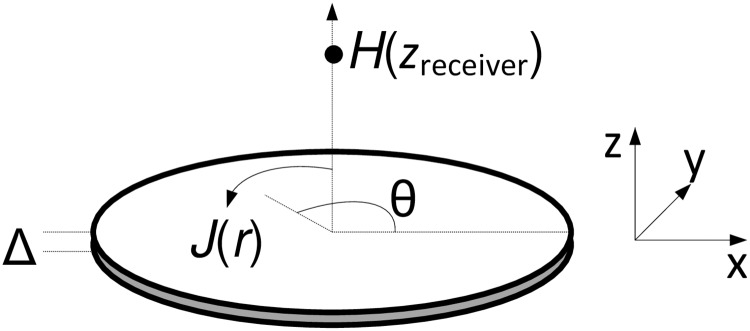
A flat disc with symmetrical current distribution *J*(*r*).

The disc is characterized by a radius *R* and a thickness of Δ, which is considered much smaller than other dimensions of interest (Fig 2). It carries a symmetrical current density *J*(*r*) in a direction perpendicular to the radius:

$$J=J\left(r\right)\hat{\theta }$$ (8)

The near axial magnetic field is derived from [2] as:

$$H_{z}=\frac{m}{2\pi z^{3}}$$ (9)

where *m* is the magnetic moment and *z* is the axial distance. Therefore, the design problem may be reformulated as:

$$\begin{array}{l} \text{min}.\;W=\frac{\mu_{0}}{4}{\underset{\begin{array}{l} \text{ all}\\ \text{space} \end{array}}{∭}\left|\vec{H}(\vec{p})\right|}^{2}dv\\ \text{s.t. }m_{disc}=\Delta \pi \int_{0}^{R}r^{2}\cdot J\left(r\right)dr=1\;{\text{Am}}^{2} \end{array}$$ (10)

where *m*_*disc*_ is the magnetic moment of the disc. For simplicity, the optimization problem is solved under normalized equality constraint of *m*_disc_ = 1 Am^2^, and the proportion constant that multiples *J*(*r*) is found after the optimal distribution is found.

The magnetic field at an arbitrary point *p* in space may be computed using the Biot-Savart Law:

$$\vec{H}(\vec{p})=\frac{\Delta }{4\pi }\int_{0}^{R}J\left(r\right)\int_{-\pi }^{+\pi }\frac{\left(-\text{sin}\theta \hat{\text{x}}\text{+cos}\theta \hat{\text{y}}\right)}{{\left|\vec{p}-r\text{cos}\theta \hat{\text{x}}-r\text{sin}\theta \hat{\text{y}}\right|}^{3}}\left(\vec{p}-r\text{cos}\theta \hat{\text{x}}-r\text{sin}\theta \hat{\text{y}}\right)rdrd\theta$$ (11)

Substituting an arbitrary point *p* in Cartesian coordinates:

$$\begin{array}{c} \vec{p}=x\hat{\text{x}}+y\hat{\text{y}}+z\hat{\text{z}}\\ \vec{H}(\vec{p})=\frac{\Delta }{4\pi }\int_{0}^{R}rJ\left(r\right)dr\int_{-\pi }^{+\pi }\frac{z\text{cos}\theta \cdot \hat{\text{x}}+z\text{sin}\theta \cdot \hat{\text{y}}+\left(r-y\text{sin}\theta -x\text{cos}\theta \right)\cdot \hat{\text{z}}}{{\left({\left(x-r\text{cos}\theta \right)}^{2}+{\left(y-r\text{sin}\theta \right)}^{2}+z^{2}\right)}^{3/2}}d\theta \end{array}$$ (12)

Substituting Eqs (12) into (10) results in the following equivalent problem:

$$\begin{array}{l} \text{min}.\;W=\frac{\mu_{0}\Delta^{2}}{{64\pi }^{2}}{\underset{\begin{array}{l} \text{ all}\\ \text{space} \end{array}}{∭}\left|\int_{0}^{R}rJ\left(r\right)dr\int_{-\pi }^{+\pi }\frac{z\text{cos}\theta \cdot \hat{\text{x}}+z\text{sin}\theta \cdot \hat{\text{y}}+\left(r-y\text{sin}\theta -x\text{cos}\theta \right)\cdot \hat{\text{z}}}{{\left({\left(x-r\text{cos}\theta \right)}^{2}+{\left(y-r\text{sin}\theta \right)}^{2}+z^{2}\right)}^{3/2}}d\theta \right|}^{2}dxdydz\\ \text{s.t. }m_{disc}=\Delta \pi \int_{0}^{R}r^{2}\cdot J\left(r\right)dr=1\;{\text{Am}}^{2} \end{array}$$ (13)

The problem is simplified by the use of cylindrical coordinates. Due to symmetry, the magnetic field *H* is independent of *ϕ* and is given as follows:

$$\vec{H}\left(\rho ,z\right)=H_{\rho }\left(\rho ,z\right)\hat{\rho }+H_{z}\left(\rho ,z\right)\hat{z}$$ (14)

To evaluate the field it is thus sufficient to calculate it at *ϕ* = 0:

$$\begin{array}{c} x=\rho \;y=0\;\Rightarrow \;\hat{\rho }=\hat{x}\\ \vec{H}\left(\rho ,z\right)=\frac{\Delta }{4\pi }\int_{0}^{R}rJ\left(r\right)dr\int_{-\pi }^{+\pi }\frac{z\text{cos}\theta \cdot \hat{\rho }+z\text{sin}\theta \cdot \hat{\text{y}}+\left(r-\rho \text{cos}\theta \right)\cdot \hat{\text{z}}}{{\left({\left(\rho -r\text{cos}\theta \right)}^{2}+{\left(r\text{sin}\theta \right)}^{2}+z^{2}\right)}^{3/2}}d\theta \end{array}$$ (15)

In this expression the field directed at *y* is given by an integral over an anti-symmetric function and is therefore zero. The resulting magnetic energy is

$$W=\frac{\mu_{0}\Delta^{2}}{{16\pi }^{2}}\int_{-\pi }^{+\pi }d\phi \int_{0}^{\infty }\rho d\rho \int_{-\infty }^{\infty }dz{\left|\int_{0}^{R}rJ\left(r\right)dr\int_{0}^{\pi }\frac{z\text{cos}\theta \cdot \hat{\rho }+\left(r-\rho \text{cos}\theta \right)\cdot \hat{\text{z}}}{{\left(\rho^{2}+r^{2}-2r\rho \text{cos}\theta +z^{2}\right)}^{3/2}}d\theta \right|}^{2}$$ (16)

Exploiting symmetry in *ϕ* and the symmetry in positive and negative values of *z*:

$$W=\frac{\mu_{0}\Delta^{2}}{2\pi }\int_{0}^{\infty }\rho d\rho \int_{0}^{\infty }dz\cdot {\left|\int_{0}^{R}rJ\left(r\right)dr\int_{0}^{\pi }\frac{z\text{cos}\theta \cdot \hat{\rho }+\left(r-\rho \text{cos}\theta \right)\cdot \hat{\text{z}}}{{\left(\rho^{2}+r^{2}-2r\rho \text{cos}\theta +z^{2}\right)}^{3/2}}d\theta \right|}^{2}$$ (17)

The optimization problem becomes:

$$\begin{array}{l} \text{min}.\;W=\frac{\mu_{0}\Delta^{2}}{4\pi }\int_{0}^{\infty }\frac{1}{\rho^{3}}d\rho \int_{0}^{\infty }dz{\left|\int_{0}^{R}\frac{b\left(r\right)}{r}dr\int_{0}^{\pi }\frac{\left(\frac{z}{\rho }\right)\text{cos}\theta \cdot \hat{\rho }+\left(\frac{r}{\rho }-\text{cos}\theta \right)\cdot \hat{\text{z}}}{{\left(1+{\left(\frac{r}{\rho }\right)}^{2}-2\left(\frac{r}{\rho }\right)\text{cos}\theta +{\left(\frac{z}{\rho }\right)}^{2}\right)}^{3/2}}d\theta \right|}^{2}\\ \text{s.t. }m_{disc}=\Delta \pi \int_{0}^{R}b\left(r\right)dr=1\;{\text{Am}}^{2} \end{array}$$ (18)

where *b*(*r*) = *r*^2^*J*(*r*). The problem in now transformed to a discrete problem by sampling *b*(*r*) over a discrete grid of *M* samples:

$$\begin{array}{l} r_{i}=i\cdot dr\;,\;dr=\frac{R}{M}\;,\;i=1\ldots M\\ b_{i}=b\left(r_{i}\right)=r_{i}{}^{2}\cdot J\left(r_{i}\right) \end{array}$$ (19)

Integration over *r* is approximated as a sum over the discrete variables *b*_*i*_:

$$\begin{array}{l} \text{min}.\;W=\frac{\mu_{0}\Delta^{2}{\left(dr\right)}^{2}}{4\pi }\int_{0}^{\infty }\frac{1}{\rho^{3}}d\rho \int_{0}^{\infty }dz{\left|\sum_{i=1}^{M}\frac{b_{i}}{r_{i}}\int_{0}^{\pi }\frac{\left(\frac{z}{\rho }\right)\text{cos}\theta \cdot \hat{\rho }+\left(\frac{r}{\rho }-\text{cos}\theta \right)\cdot \hat{\text{z}}}{{\left(1+{\left(\frac{r}{\rho }\right)}^{2}-2\left(\frac{r}{\rho }\right)\text{cos}\theta +{\left(\frac{z}{\rho }\right)}^{2}\right)}^{3/2}}d\theta \right|}^{2}\\ \text{s.t. }\sum_{i=1}^{M}b_{i}=\frac{1}{\Delta \pi dr} \end{array}$$ (20)

The Lagrange multipliers method provides a sufficient condition for an extremum point:

$$\begin{array}{l} \frac{\partial W}{\partial b_{j}}+\lambda \frac{\partial }{\partial b_{j}}\left(\sum_{i=1}^{M}b_{i}-\frac{1}{\Delta \pi dr}\right)=0\;\\ \Rightarrow \;\frac{\partial W}{\partial b_{j}}=-\lambda =\text{constant} \end{array}$$ (21)

To carry out the derivation, the following functions are defined:

$$\alpha_{\rho }\left(\frac{r_{i}}{\rho },\frac{z}{\rho }\right)=\int_{0}^{\pi }d\theta \frac{\left(\frac{z}{\rho }\right)\text{cos}\theta }{{\left(1+{\left(\frac{r_{i}}{\rho }\right)}^{2}-2\left(\frac{r_{i}}{\rho }\right)\text{cos}\theta +{\left(\frac{z}{\rho }\right)}^{2}\right)}^{3/2}}$$ (22)

$$\alpha_{z}\left(\frac{r_{i}}{\rho },\frac{z}{\rho }\right)=\int_{0}^{\pi }d\theta \frac{\left(\frac{r_{i}}{\rho }-\text{cos}\theta \right)}{{\left(1+{\left(\frac{r_{i}}{\rho }\right)}^{2}-2\left(\frac{r_{i}}{\rho }\right)\text{cos}\theta +{\left(\frac{z}{\rho }\right)}^{2}\right)}^{3/2}}$$ (23)

This simplifies the energy function as follows:

$$W=\frac{\mu_{0}\Delta^{2}{\left(dr\right)}^{2}}{4\pi }\int_{0}^{\infty }\frac{1}{\rho^{3}}d\rho \int_{0}^{\infty }dz{\left|\sum_{i=1}^{M}\frac{b_{i}}{r_{i}}\left(\alpha_{\rho }\left(\frac{r_{i}}{\rho },\frac{z}{\rho }\right)\cdot \hat{\rho }+\alpha_{z}\left(\frac{r_{i}}{\rho },\frac{z}{\rho }\right)\cdot \hat{\text{z}}\right)\right|}^{2}$$ (24)

The condition for optimality becomes:

$$\frac{\partial }{\partial b_{j}}\{\int_{0}^{\infty }\frac{1}{\rho^{3}}d\rho \int_{0}^{\infty }dz\cdot |\sum_{i=1}^{M}\frac{b_{i}}{r_{i}}(\alpha_{\rho }\left(\frac{r_{i}}{\rho },\frac{z}{\rho }\right)\cdot \hat{\rho }{+\alpha_{z}\left(\frac{r_{i}}{\rho },\frac{z}{\rho }\right)\cdot \hat{\text{z}})}^{2}|=\lambda \prime =\text{constant}$$ (25)

For every *j* = 1…*M*:

$$\frac{\partial }{\partial b_{j}}\{\int_{0}^{\infty }\frac{1}{\rho^{3}}d\rho \int_{0}^{\infty }{\left(\sum_{i=1}^{M}\frac{b_{i}}{r_{i}}\alpha_{\rho }\left(\frac{r_{i}}{\rho },\frac{z}{\rho }\right)\right)}^{2}+{\left(\sum_{i=1}^{M}\frac{b_{i}}{r_{i}}\alpha_{z}\left(\frac{r_{i}}{\rho },\frac{z}{\rho }\right)\right)}^{2}dz\}=\lambda \prime =\text{constant}$$ (26)

Exchanging derivation and integration:

$$\begin{array}{l} \int_{0}^{\infty }\frac{1}{\rho^{3}}d\rho \int_{0}^{\infty }(\frac{\partial }{\partial b_{j}}{\left(\sum_{i=1}^{M}\frac{b_{i}}{r_{i}}\alpha_{\rho }\left(\frac{r_{i}}{\rho },\frac{z}{\rho }\right)\right)}^{2}+\frac{\partial }{\partial b_{j}}{\left(\sum_{i=1}^{M}\frac{b_{i}}{r_{i}}\alpha_{z}\left(\frac{r_{i}}{\rho },\frac{z}{\rho }\right)\right)}^{2})dz=\lambda \prime =\text{const.}\\ \int_{0}^{\infty }\frac{1}{\rho^{3}}d\rho \int_{0}^{\infty }(\left(\sum_{i=1}^{M}\frac{b_{i}}{r_{i}}\alpha_{\rho }\left(\frac{r_{i}}{\rho },\frac{z}{\rho }\right)\right)\frac{\alpha_{\rho }\left(\frac{r_{j}}{\rho },\frac{z}{\rho }\right)}{r_{j}}+\left(\sum_{i=1}^{M}\frac{b_{i}}{r_{i}}\alpha_{z}\left(\frac{r_{i}}{\rho },\frac{z}{\rho }\right)\right)\frac{\alpha_{z}\left(\frac{r_{j}}{\rho },\frac{z}{\rho }\right)}{r_{j}})dz=\lambda \prime \prime =\text{const.} \end{array}$$ (27)

Exchanging the order of sum and integral:

$$\sum_{i=1}^{M}b_{i}\frac{1}{r_{i}r_{j}}\cdot \int_{0}^{\infty }\int_{0}^{\infty }\frac{1}{\rho^{3}}(\alpha_{\rho }\left(\frac{r_{i}}{\rho },\frac{z}{\rho }\right)\alpha_{\rho }\left(\frac{r_{j}}{\rho },\frac{z}{\rho }\right)+\alpha_{z}\left(\frac{r_{i}}{\rho },\frac{z}{\rho }\right)\alpha_{z}\left(\frac{r_{j}}{\rho },\frac{z}{\rho }\right))dzd\rho =\lambda \prime \prime =\text{const.}$$ (28)

Define the discrete variables *u*_*j*, *i*_:

$$u_{j,i}=\frac{1}{r_{i}r_{j}}\int_{0}^{\infty }\int_{0}^{\infty }\frac{1}{\rho^{3}}(\alpha_{\rho }\left(\frac{r_{i}}{\rho },\frac{z}{\rho }\right)\alpha_{\rho }\left(\frac{r_{j}}{\rho },\frac{z}{\rho }\right)+\alpha_{z}\left(\frac{r_{i}}{\rho },\frac{z}{\rho }\right)\alpha_{z}\left(\frac{r_{j}}{\rho },\frac{z}{\rho }\right))dzd\rho$$ (29)

Define the matrix *U*_*MxM*_:

$$U=\left(\begin{array}{ccc} u_{1,1} & \cdots & u_{1,M}\\ \vdots & \ddots & \vdots \\ u_{M,1} & \cdots & u_{M,M} \end{array}\right)$$ (30)

Eq (28) may be expressed in matrix notation:

$$U\cdot \left(\begin{array}{c} b_{1}\\ \vdots \\ b_{M} \end{array}\right)=\lambda \prime \prime \left(\begin{array}{c} 1\\ \vdots \\ 1 \end{array}\right)\;\Rightarrow \;\left(\begin{array}{c} b_{1}\\ \vdots \\ b_{M} \end{array}\right)=\left(\lambda \prime \prime \right)\cdot U^{-1}\cdot \left(\begin{array}{c} 1\\ \vdots \\ 1 \end{array}\right)$$ (31)

The equation *b*_*i*_ = *J*(*r*_*i*_)∙*r*_*i*_^2^ leads to the following expression:

$$\left(\begin{array}{c} J\left(r_{1}\right)\\ \vdots \\ J\left(r_{M}\right) \end{array}\right)=\left(\lambda \prime \prime \right)\cdot \left(\begin{array}{ccc} \frac{1}{r_{1}{}^{2}} & & 0\\ & \ddots & \\ 0 & & \frac{1}{r_{M}{}^{2}} \end{array}\right)\cdot U^{-1}\cdot \left(\begin{array}{c} 1\\ \vdots \\ 1 \end{array}\right)$$ (32)

This is the optimal current distribution. The factor *λ*″ is chosen to provide the desired axial magnetic field *H*(*z*). Once the current distribution *J*(*r*) is found, the magnetic field in space may be computed using the precomputed function *α*_*p*_ and *α*_*z*_:

$$\vec{H}\left(\rho ,z\right)=\frac{\Delta }{2\pi \rho^{2}}\int_{0}^{R}rJ\left(r\right)\left(\alpha_{\rho }\left(\frac{r}{\rho },\frac{z}{\rho }\right)\cdot \hat{\rho }+\alpha_{z}\left(\frac{r}{\rho },\frac{z}{\rho }\right)\cdot \hat{\text{z}}\right)dr$$ (33)

This method provides a current profile that can be well approximated as follows:

$$J\left(r\right)=K\left(\sqrt{\frac{R}{R-\alpha r}}+\frac{r}{R}-1\right)$$ (34)

Where *α* approaches unity. The constant *K* should be chosen to provide the required magnetic field at a distant receiver. This varying current density can be realized using varying number of turns wound at specific radius.

## Minimal reactive power in general 3D transmitter coils

This section extends the previous result to general 3D coil topologies. In this problem the location of the current loops within the transmitter are predefined, and the objective is to calculate the optimal amplitudes. This enables an analytic solution for general coil geometries.

The coil is modeled as a system of coupled single-turn inductors. Each inductor carries a current *i*_*i*_ and has an area *A*_*i*_. The problem of reactive power minimization under constraint of required magnetic field at the receiver is formulated as

$$\begin{array}{l} \text{min}:\;W=\frac{1}{2}I^{T}MI\\ \text{s.t. }H_{z}\left(z_{receiver}\right)=\sum_{j=1}^{N}\frac{A_{j}i_{j}}{2\pi z^{3}}=\frac{A^{T}I}{2\pi z_{receiver}^{3}} \end{array}$$ (35)

Where *I* is a column vector consisting of currents *i*_*i*_, *A* is a column vector, consisting of *A*_*i*_, *M* is an inductance matrix composed of self and mutual elements, and *H*_*z*_(*z*_*receiver*_) is the near magnetic field magnitude at the receiver location on the z axis [2]. Applying the Lagrange Multipliers method, a Lagrangian is defined:

$$l=\frac{1}{2}I^{T}MI-\lambda \left(A^{T}I-2\pi z_{receiver}^{3}H\left(z_{receiver}\right)\right)$$ (36)

Using matrix derivation rules, the following derivatives are obtained

$$\begin{array}{l} \frac{\partial l}{\partial I}=\left(\begin{array}{c} \frac{\partial l}{\partial i_{1}}\\ \vdots \\ \frac{\partial l}{\partial i_{N}} \end{array}\right)=\frac{1}{2}\left(M+M^{T}\right)I+\lambda A\;\\ \frac{\partial l}{\partial \lambda }=A^{T}I-2\pi z_{receiver}^{3}H\left(z_{receiver}\right) \end{array}$$ (37)

Equating the derivatives to zero, the following set of linear equations is obtained

$$\begin{array}{l} MI+\lambda A=0\\ A^{T}I-2\pi z_{receiver}^{3}H\left(z_{receiver}\right)=0 \end{array}$$ (38)

Where we have used the symmetry of the matrix, *M* = *M*^T^. By substituting the first equation into the second

$$\begin{array}{l} I=-\lambda M^{-1}A\;\Rightarrow \;\\ A^{T}\left(-\lambda M^{-1}A\right)=2\pi z_{receiver}^{3}H\left(z_{receiver}\right) \end{array}$$ (39)

This leads to a solution for λ

$$\lambda =-\frac{2\pi z_{receiver}^{3}H\left(z_{receiver}\right)}{A^{T}M^{-1}A}$$ (40)

The resulting current vector is given by

$$I=2\pi z_{receiver}^{3}H\left(z_{receiver}\right)\frac{M^{-1}A}{A^{T}M^{-1}A}$$ (41)

This expression reveals an analytic expression for the optimal preselected inductor currents that minimizes the reactive power.

## Simulation results demonstrating minimal reactive power

This subsection presents the results of the two proposed optimization methods. We show the optimal current distributions for several coil geometries, including a flat coil, multi-layer solenoid, and a single layer solenoid. For the flat coil geometry the two methods are shown to provide similar results.

**Fig 3:**
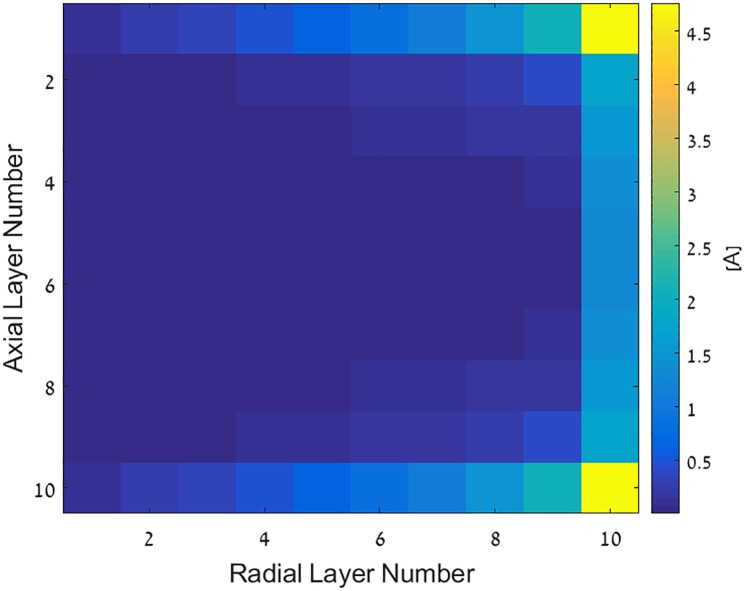
Current distribution in optimal multi-layer solenoid coil.

For multi-layer coils, the optimal current distribution is computed according to Eq (41). The inductance matrix *M* is computed semi-analytically, employing elliptic integrals, and is cross checked with numerical method in [22] to validate accuracy. The parameters of the considered coil are as follows: the outer and inner radii are 10 and 5 cm, respectively. The number of turns in both the radial and axial directions is 10, and the turns are equally spaced in radial and axial directions, respectively. The coil height is 2 cm, and the wire radius is 1 mm. Fig 3 shows the current distribution at cross section of this multi-layer coil; only one-half of the cross section is shown due to symmetry. The coil is oriented along y-axis, hence the x-axis of Fig 3 presents the current distribution along the radial dimension of the coil, and y-axis presents the current distribution along the axis of the coil.

Results show that the desired current magnitude rises towards the external turns, both radially and axially. Practically, this varying current density can be achieved by winding the wire several times at a specific radius and height.

**Fig 4:**
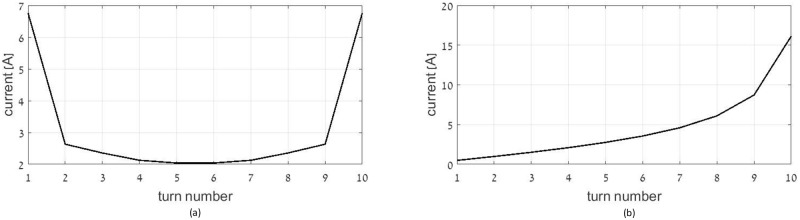
Current distribution in (a) the optimal single layer solenoid coil, and (b) the optimal spiral flat coil.

The current distribution of an optimal single layer solenoid with all the other parameters being identical to the previously considered multi-layer coil is depicted in Fig 4(a). The desired current magnitudes increase towards the external turns of the coil. Fig 4(b) shows the current distribution of an optimal flat coil with all the other parameters being identical to the previously considered multi-layer coil. As expected from the two previous cases, the current magnitudes increase towards external layers.

**Fig 5:**
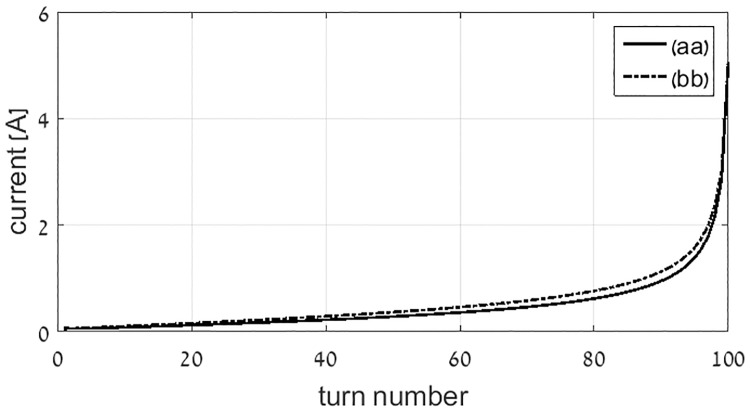
Optimal current comparison of the flat coil with (aa) the predefined current loop locations method with that of (bb) the non-predefined current loop locations method.

Next, a comparison between the predefined and the non-predefined current loop locations methods is shown for the flat coil with large number of turns. As can be seen in Fig 5, both current distributions nearly coincide. The coil parameters are as follows: the outer and the inner radii are 10 cm and 0.1 cm, respectively, and the number of turns is 100. A *α* coefficient of the formula Eq (34) is 0.995, and this coefficient approaches unity as number of turns increase.

To evaluate the reactive power reduction obtained by the optimal coil design we compare various coil topologies designed by the presented method under the constraint of identical magnetic field *A*^*T*^*I* = 1. We show the relative advantage of the various topologies based on constraints imposed on the system design as well as possible cost and weight savings.

**Fig 6:**
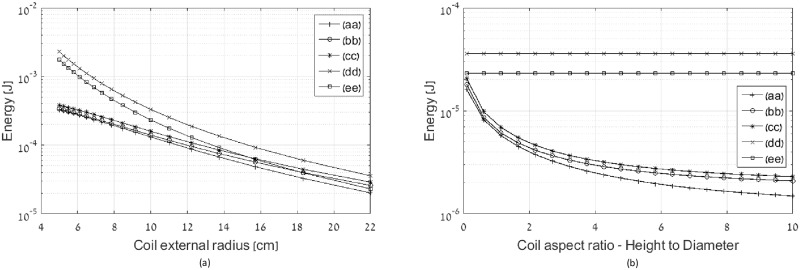
(a) Energy stored at various coil topologies with constraint of constant volume and weight, (b) energy stored at various coil topologies as function of aspect ratio with constraint of constant number of turns. The energy stored in flat coils having the same radius are shown for reference only. Legend entries: (aa) Optimal current distribution multi-layer solenoid coil, (bb) Optimal current distribution single layer solenoid coil, (cc) Constant current single layer solenoid coil, (dd) Constant current single layer flat coil, and (ee) Optimal current distribution spiral flat coil.

First we show energy stored in the transmitting coil under the constraint of constant volume and constant weight. The external radius of the coil is varied between 5 and 22 cm, the height is simultaneously varied between 19.36 and 1 cm to keep the constant volume, number of turns in axial direction is varied between 22 and 5 to keep the constant weight, and number of turns in radial direction is 10. The resultant stored energy is shown in Fig 6(a) for the following topologies: (aa) Optimal current distribution multi-layer solenoid coil, (bb) Optimal current distribution single layer solenoid coil, (cc) Constant current single layer solenoid coil, (dd) Constant current single layer flat coil, and (ee) Optimal current distribution spiral flat coil. The flat coil topologies are shown for reference, as they do not keep the constant volume and weight constraint of their 3D counterparts.

Two main conclusions can be inferred from observing Fig 6(a). The first one is that for high aspect ratios (height to diameter) of the coil, the energy stored in various 3D coils practically coincides, and no benefit is offered by optimal current distribution. However, flat coils with the same radii as the high aspect ratio coils, are inferior. The second conclusion is that reduction of the aspect ratio, under the constraint of keeping constant volume and constant weight, is beneficial in terms of stored energy, and the optimal flat spiral coil approaches the optimal multi-layer coil. Yet, the constant current single layer flat coil is substantially inferior to the optimal flat spiral coil.

Next we explore effect of pitch increases. This reduces the coupling between turns, thus reducing the coil inductance. Fig 6(b) illustrates the benefit of aspect ratio increase for all 3D topologies. The coil parameters are as follows: external radius is 22 cm, number of turns in axial direction is 20, and number of turns in radial direction at multi-layer coils is 10. Energy stored in the flat topologies having the same external radius and number of turns in radial direction is shown for reference.

**Fig 7:**
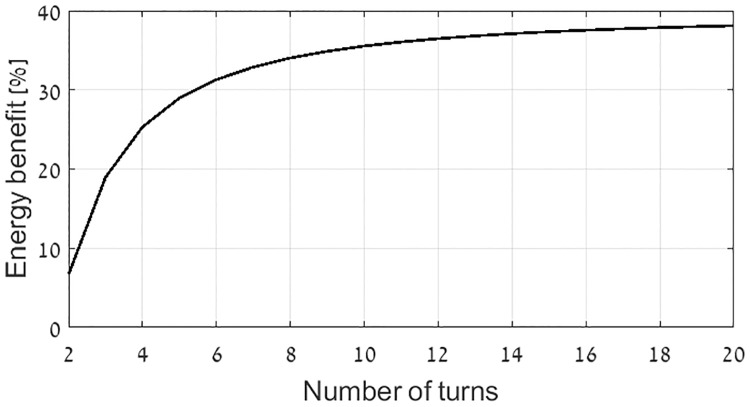
Energy benefit of optimal spiral flat coil over constant current single layer flat coil.

The benefit in terms of energy of the optimal flat spiral coil in comparison to constant current single layer flat coil is shown in Fig 7, as a function of the number of turns of the optimal coil. The external radii of the coils considered in Fig 7 are 22 cm, the interior radius of the optimal spiral coil is 1/2 of its external radius, and its number of turns is varied between 2 to 20. It can be inferred from the graph that optimal spiral flat coil is substantially better than the constant current single layer flat coil.

**Fig 8:**
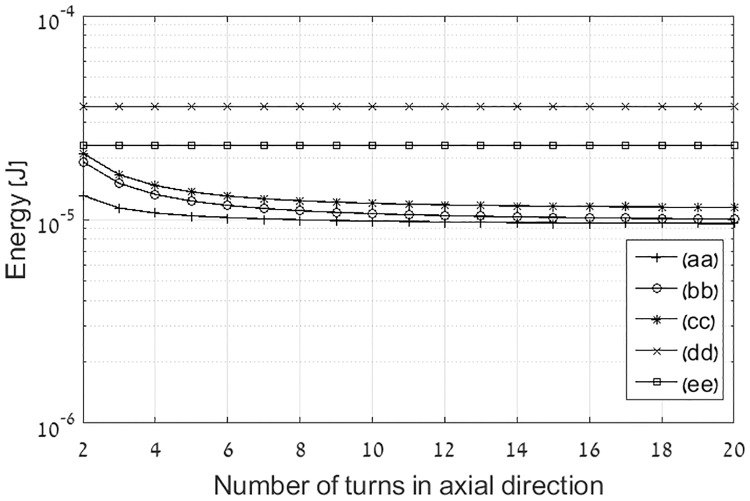
Energy stored at various coils topologies as function of number of turns in axial direction with constraint of constant dimensions. The energy stored in flat coils having the same radius are shown for reference only. Legend entries: (aa) Optimal current distribution multi-layer solenoid coil, (bb) Optimal current distribution single layer solenoid coil, (cc) Constant current single layer solenoid coil, (dd) Constant current single layer flat coil, and (ee) Optimal current distribution spiral flat coil.

At the last stage we explore the impact of number of turns with a given coil dimensions. Fig 8 shows the energy stored in various coil topologies under the constraint of constant exterior dimensions. The coil parameters are as follows: external radius is 22 cm, number of turns in axial direction is varied between 2 and 20, number of turns in radial direction at multi-layer coils is 10, and the coil height is 20 cm. Energy stored in the flat topologies having the same external radius and number of turns in radial direction is shown for reference. It can be inferred from observing Fig 8 that a decrease in the number of turns in axial direction, only slightly increases the stored energy, yet it provides weight and cost benefits.

## Minimal apparent power in general 3D transmitter coils

This section extends the previous analysis by considering, in addition to reactive power, the active power losses in the transmitter coil. Also, under consideration, is the total apparent power consumption. This compound objective is significant in weakly coupled non-radiating systems, since the total apparent power determines the peak instantaneous power required from the transmitter circuitry, and the active power is associated with resistive losses, and determines the transmitter efficiency. The following analysis provides a low complexity numeric solution method, which converts the original high dimensional problem to an equivalent problem with two free variables.

The transmitting coil is viewed as a system of coupled inductors, in which the terminal voltages *V* and the total coil power *S* are expressed by

$$\begin{array}{l} V=Mj\omega I+RI\\ S=I^{*}V \end{array}$$ (42)

where *I* is a vector of currents, *V* is a vector of voltages, *M* is the inductance matrix that includes the self and the mutual elements, and *R* is the diagonal resistance matrix, calculated by [23]. The magnitude of the apparent power equals:

$$|S{|}^{2}=|I^{*}(Mj\omega I+RI){|}^{2}=|I^{*}Mj\omega I+I^{*}RI{|}^{2}=|j\omega I^{*}MI+I^{*}RI{|}^{2}$$ (43)

Assuming the reference phase in the system is that of the coil, and setting all currents in phase, the apparent power magnitude results in:

$$|S{|}^{2}=|j\omega I^{T}MI+I^{T}RI{|}^{2}={\left(I^{T}\omega MI\right)}^{2}+{\left(I^{T}RI\right)}^{2}$$ (44)

The optimization goal is minimization of the apparent power under the constraint of a given axial near field:

$$\begin{array}{l} \text{min }|S{|}^{2}\\ \text{s.t. }A^{T}I=1 \end{array}$$ (45)

Where *A* is the areas column vector.

The Lagrangian is defined by

$$\begin{array}{l} L=|S{|}^{2}+\lambda \left(A^{T}I-1\right)\\ \frac{\partial L}{\partial I_{i}}=0,\text{ for all }i\\ \frac{\partial L}{\partial \lambda }=0\to A^{T}I=1 \end{array}$$ (46)

The derivative of |*S*|^2^ with respect to currents:

$$2\omega^{2}I^{T}MI\left(M+M^{T}\right)I+2I^{T}RI\left(R+R^{T}\right)I+\lambda A=0$$ (47)

The *M* and *R* matrix are symmetric:

$$\left\{ \begin{array}{l} 4\omega^{2}I^{T}MIMI+4I^{T}RIRI+\lambda A=0\\ A^{T}I=1 \end{array}\right.$$ (48)

Auxiliary variables are defined to simplify the solution of Eq (48):

$$\left\{ \begin{array}{l} C=4\omega^{2}I^{T}MI\\ D=4I^{T}RI \end{array}\right.$$ (49)

Which results in the following system of equations:

$$\left\{ \begin{array}{l} CMI+DRI+\lambda A=0\\ {\text{A}}^{T}I=1\\ C=4\omega^{2}I^{T}MI\\ D=4I^{T}RI \end{array}\right.\;$$ (50)

$$I=-\lambda {\left(\text{CM}+\text{DR}\right)}^{-1}A$$ (51)

$$A^{T}\left(-\lambda {\left(\text{CM}+\text{DR}\right)}^{-1}A\right)=1$$ (52)

$$\lambda =\frac{-1}{A^{T}{\left(\text{CM}+\text{DR}\right)}^{-1}A}$$ (53)

$$I=\frac{{\left(\text{CM}+\text{DR}\right)}^{-1}A}{A^{T}{\left(\text{CM}+\text{DR}\right)}^{-1}A}$$ (54)

The resultant systems of equations:

$$\left\{ \begin{array}{l} I=\frac{{\left(\text{CM}+\text{DR}\right)}^{-1}A}{A^{T}{\left(\text{CM}+\text{DR}\right)}^{-1}A}\\ {\text{A}}^{T}I=1\\ C=4\omega^{2}I^{T}MI\\ D=4I^{T}RI \end{array}\right.$$ (55)

This system of equations is checked at *R* = 0 for consistency with Eq (41):

$$\left\{ \begin{array}{l} I=\frac{{\left(\text{CM}\right)}^{-1}A}{A^{T}{\left(\text{CM}\right)}^{-1}A}=\frac{C^{-1}M^{-1}}{C^{-1}A^{T}M^{-1}A}=\frac{M^{-1}}{A^{T}M^{-1}A}\\ C=4\omega^{2}I^{T}MI \end{array}\right.$$ (56)

which is identical to current distribution with reactive power limit.

Substituting the first equation of Eq (55) into the third and fourth of Eq (55):

$$\left\{ \begin{array}{l} C=4\omega^{2}{\left(\frac{{\left(\text{CM}+\text{DR}\right)}^{-1}A}{A^{T}{\left(\text{CM}+\text{DR}\right)}^{-1}A}\right)}^{T}M\frac{{\left(\text{CM}+\text{DR}\right)}^{-1}A}{A^{T}{\left(\text{CM}+\text{DR}\right)}^{-1}A}\\ D=4{\left(\frac{{\left(\text{CM}+\text{DR}\right)}^{-1}A}{A^{T}{\left(\text{CM}+\text{DR}\right)}^{-1}A}\right)}^{T}R\frac{{\left(\text{CM}+\text{DR}\right)}^{-1}A}{A^{T}{\left(\text{CM}+\text{DR}\right)}^{-1}A} \end{array}\right.$$ (57)

Due to the fact that *M* and *R* are symmetric matrices:

$$\left\{ \begin{array}{l} C=4\omega^{2}\frac{A^{T}{\left(\text{CM}+\text{DR}\right)}^{-1}M{\left(\text{CM}+\text{DR}\right)}^{-1}A}{{\left(A^{T}{\left(\text{CM}+\text{DR}\right)}^{-1}A\right)}^{2}}\\ D=4\frac{A^{T}{\left(\text{CM}+\text{DR}\right)}^{-1}R{\left(\text{CM}+\text{DR}\right)}^{-1}A}{{\left(A^{T}{\left(\text{CM}+\text{DR}\right)}^{-1}A\right)}^{2}} \end{array}\right.$$ (58)

The last system of equations is numerically solved for *C* and *D*, and then the current distribution is found using Eq (55). For computing the power and losses, the resistance matrix *R* is adjusted to match the actual current distribution.

## Simulation results demonstrating minimal apparent power

**Fig 9:**
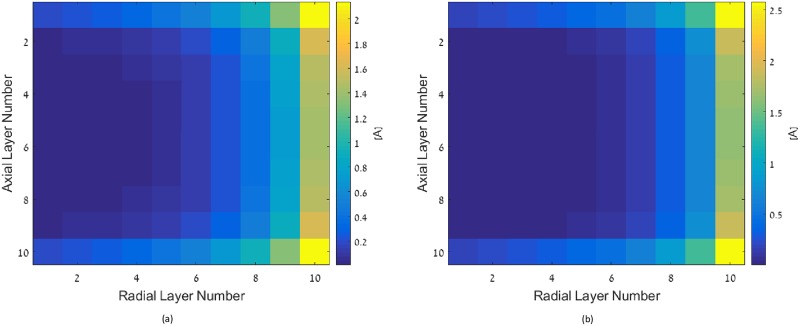
Current distribution in optimal multi-layer solenoid coil: wire radius: (a) 0.1 mm, (b) 0.5 mm.

**Fig 10:**
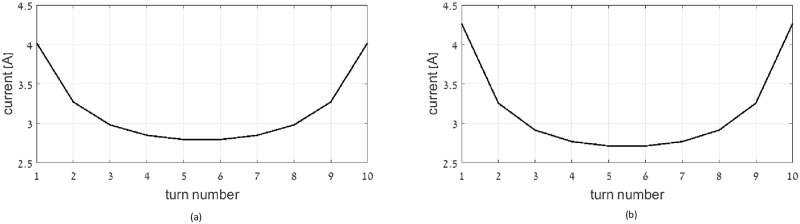
Current distribution in optimal single-layer solenoid coil: wire radius: (a) 0.1 mm, (b) 0.5 mm.

**Fig 11:**
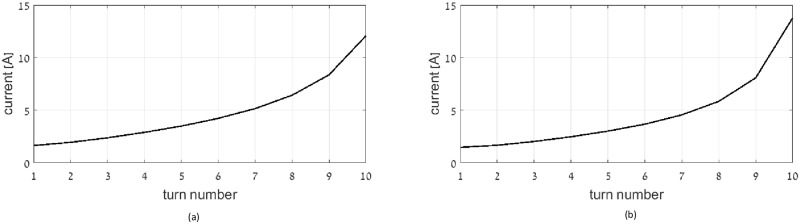
Current distribution in optimal flat coil: wire radius: (a) 0.1 mm, (b) 0.5 mm.

This section demonstrates the optimal current distribution that minimizes total apparent power. The considered coil dimensions are similar to that considered in the reactive power optimization section. Figs 9, 10 and 11 show the current distribution of the optimal multi-layer coil, optimal single layer solenoid and optimal flat coil for (a) wire radius of 0.1 mm, and (b) 0.5 mm, respectively.

When considering the impact of wire diameter on the resultant current distribution, the results show that current distribution corresponding to thinner wires is more uniform between inner and outer turns. This current profile distributes the electric currents more evenly among larger number of loops, and thus reduces the resistive losses.

Comparing the current distribution obtained under reactive power limit with that obtained under apparent power limit, it can be inferred that at the lossless case the current gradient is sharper, compared to the lossy case.

**Fig 12:**
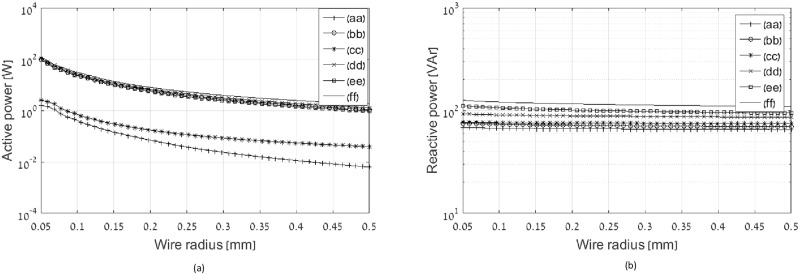
Active and reactive power as function of wire radius. Legend entries: (aa) optimal multi-layer coil, (bb) optimal single layer solenoid coil, (cc) constant current multi-layer coil, (dd) constant current single layer solenoid coil, (ee) optimal flat coil, and (ff) constant current flat coil.

To evaluate the benefit of the optimal current distribution, the active and reactive power results are shown, under the constraint of axial near field normalization: *A*^*T*^*I* = 1. First, the impact of wire radius is explored, as shown at Fig 12. It can be inferred from Fig 12 that optimal multi-layer coil is significantly superior to all other topologies for every wire diameter.

**Fig 13:**
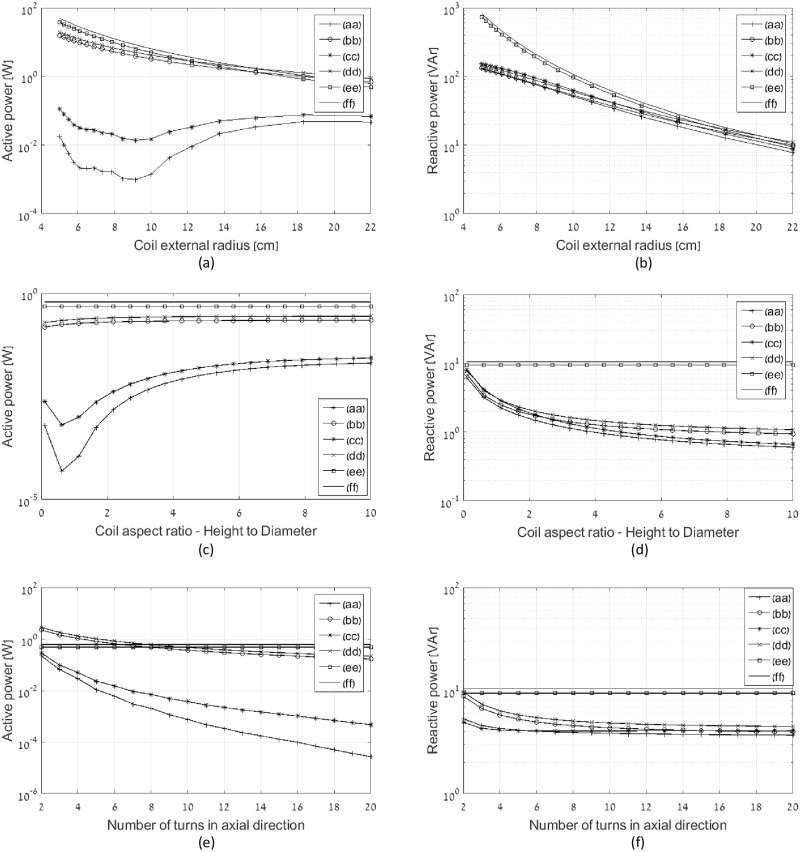
Active and reactive power of various coil topologies under number of constraints: (a) and (b) constant volume and weight, (c) and (d) constant external radius and weight, (e) and (f) constant coil dimensions. Legend entries: (aa) optimal multi-layer coil, (bb) optimal single layer solenoid coil, (cc) constant current multi-layer coil, (dd) constant current single layer solenoid coil, (ee) optimal flat coil, and (ff) constant current flat coil.

The following simulations are done at wire radius of 0.25 mm. Fig 13 show active and reactive power of the transmitting coil under variety of constraints. The constraint in Fig 13(a) and 13(b) is the constant volume and constant weight. The external radius of the coil is varied between 5 and 22 cm, the height is simultaneously varied between 19.36 and 1 cm to keep the constant volume, number of turns in axial direction is varied between 22 and 5 to keep the constant weight, and number of turns in radial direction is 10. Fig 13(c) and 13(d) illustrate the impact of aspect ratio increase keeping constant external radius and weight. The coils parameters are as follows: external radius is 22 cm, number of turns in axial direction is 20, and number of turns in radial direction at multi-layer coils is 10. Active and reactive power of the flat topologies having the same external radius and number of turns in radial direction is shown for reference. Fig 13(e) and 13(f) explore the impact of number of turns in given coil dimensions. The coils parameters are as follows: external radius is 22 cm, number of turns in axial direction is varied between 2 and 20, number of turns in radial direction at multi-layer coils is 10, and the coil height is 20 cm. Active and reactive power of the flat topologies having the same external radius and number of turns in radial direction is shown for reference. At all cases shown in Fig 13, the optimal multi-layer coil is significantly superior to other topologies at active power, and all 3D coils are vastly superior to flat coils at reactive power.

## Geometrical limits of the proposed approach

**Fig 14:**
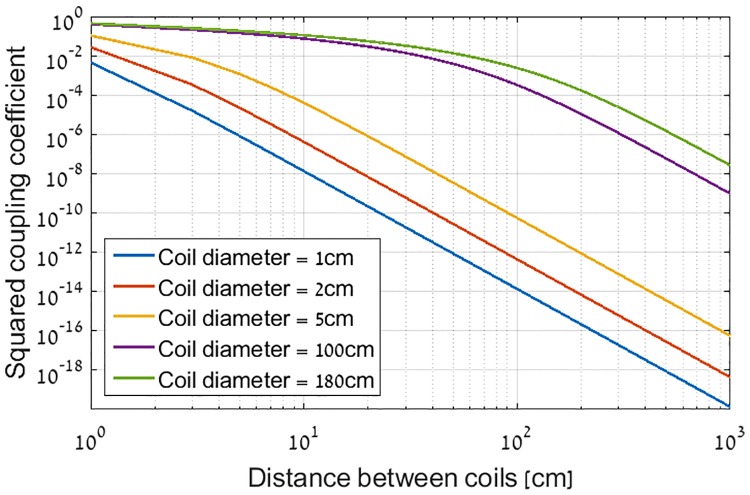
Maximum coupling coefficient of various coaxial coils as function of distance between them.

An underlying assumption of the proposed approach is that the transmitter and receiver coils are weakly coupled, so the transmitter and the receiver can be optimized independently. This section shows typical conditions in which this assumption is valid. In the general case the transmitter cannot be designed independently of the receiver, due to the reflected impedance which is proportional to the square of the coupling coefficient (*k*^2^). However, as shown in Fig 14, the roll-off rate of coupling with distance is very steep. For small coils that can be used for BAN communication (we assume a diameter below 5 cm) it can be seen that the squared coupling coefficient drops to 10^−4^ at distance of 8 cm. For Through-the-Earth mines communication applications, larger coils are used, up to 1.8 m diameter [24]. For such coil the squared coupling coefficient drops below 10^−4^ at distance of 2.2 m, while the communication range is in order of hundreds of meters. With such low coupling coefficient levels, the transmitter and receiver are virtually decoupled, and hence, the reflected impedance can be neglected.

## Receiver design considerations

The receiving antenna may be designed based on several principles. For instance, increased sensitivity of magnetic receivers can be obtained by optimizing the receiver coils structure and circuitry, as proposed in [25–30]. These works do not focus specifically on MI communication, and mainly consider search coil magnetometers, but may also enable range extension of weakly coupled MI communication systems.

In case the receiver design objective is optimal channel capacity (which might be different from optimal sensitivity), the method proposed in this paper may be applicable. To this end, we suggest to design a receiver that minimizes the reactive power for a required magnetic field. As a result, the coil inductance and quality factor of the resonant circuit are minimized for total received magnetic flux, and the bandwidth and channel capacity are maximized.

According to Eq (6), by reducing the ratio of the reactive power to desired magnetic field at the certain distance, the ratio of inductance to effective area is reduced as well. Thus, by reducing the inductance per effective area the quality factor decreases. The capacity as a function of the quality factor is shown in Eqs (59) and (60). According to [16], assuming a worst case SNR of unity over a frequency band around *f*_0_, the capacity is given by

$$C=B_{f}f_{0}{\text{log}}_{2}\left(1+SNR\right)=B_{f}f_{0}{\text{log}}_{2}\left(2\right)=B_{f}f_{0}$$ (59)

Where *B*_*f*_ = min{1/*Q*_*tx*_, 1/*Q*_*rx*_} is the fractional bandwidth, and *Q*_*tx*_, *Q*_*rx*_ are the quality factors of the transmitter and receiver. The resulting capacity is inversely proportional to quality factor

$$C=f_{0}/\text{max}\left\{Q_{tx},Q_{rx}\right\}$$ (60)

Therefore, the receiver designed according the reactive power minimization method maximizes the channel capacity.

## Experimental results

**Fig 15:**
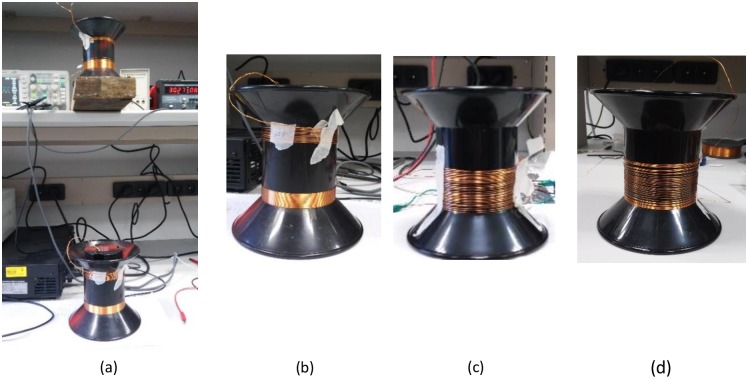
(a) An experimental setup, showing the magnetic transmitter and receiver coils. Here, the transmitter is 30 turn coil with minimal pitch. The receiver is 30 turn coil located 50 cm above the transmitter. (b) Transmitting coils for case (aa) 30 turn minimal pitch coil, and for case (bb) 15 turn increased pitch coil. (c) Transmitting coil for case (cc) 30 turn increased pitch coil. (d) Optimal current distribution single layer solenoid coil.

A series of experiments was conducted to validate the theoretical results presented above. The transmitting coil was placed on the desk and excited by 30 kHz sine wave source, while the receiving coil was located 50 cm above the transmitter. The open circuit voltage induced in the receiving coil was measured by an oscilloscope. The experimental setup is shown in Fig 15(a). The experiments were conducted under the constraint of identical magnetic field at the receiver, with various transmitting coils.

The first experiment validated weight and cost saving. Single layer solenoids were wound on a bobbin with diameter of 9 cm. The wire diameter was 0.554 mm. Three coils were evaluated: (aa) 30 turn with close pitch, resulting in coil height of h = 1.7 cm, (bb) 15 turn with increased pitch, so the coil height is similar to that of (aa)–h = 1.7 cm, both shown in Fig 15(b), and (cc) 30 turn coil with increased pitch, resulting in height h = 4.5 cm, shown in Fig 15(c). Under the requirement of identical magnetic field at the receiver, the reactive power of case (aa) was 159 VAr, case (bb) 150 VAr, and case (cc) 86 VAr. The lower reactive power of case (bb) compared to (aa) stems from not exactly identical height of case (cc), but slightly higher.

**Fig 16:**
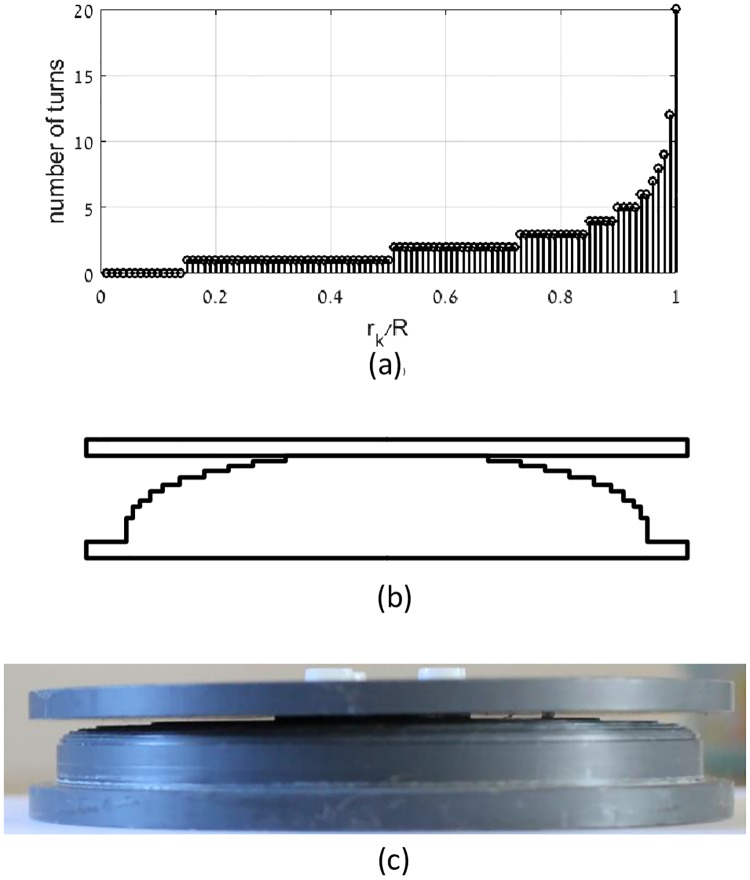
(a) Current distribution at the stepped coil, (b) the cross section sketch of the stepped coil bobbin (not to scale), and (c) the bobbin that implements the stepped current distribution.

The next experiment validated the benefit of the optimal current density flat coil over constant current flat coil. The optimal current density flat coil and a constant current flat coil with identical exterior dimensions were manufactured and evaluated. The current density of the optimal flat coil was approximated by discrete steps. At each step, the wire was wound with different number of layers to produce the total required current at given radius. The chosen quantization of current density of the stepped coil, based on rounding of Eq (34), is shown in Fig 16(a), and the cross section sketch of the stepped coil bobbin is shown in Fig 16(b) (not to scale). The bobbin, depicted in Fig 16(c), was produced using two PVC discs connected together: a stepped disc and a flat disc. The outer diameter of the stepped coil was 11 cm. The constant current flat coil was wounded on top of the stepped coil. The reactive power of the optimal current density flat coil was 112 VA, while that of the constant current flat coil was 160 VA under equal magnetic field at the receiver. Thus the optimal flat coil obtained 30% reduction in required reactive power in comparison to the constant current flat coil. Consequently, the experimental results well match the theoretical calculations.

The last experiment verified the active power reduction obtained with the optimal current distribution coil. An optimal single layer coil (Fig 15(d)) was wound and its power consumption was compared to the coil with identical dimensions and number of turns (Fig 15(c)). The power consumption of the optimal coil was 2.56 W, which is 25% lower than the power of the constant current coil, which consumed 3.2 W.

## Conclusions

Two important challenges in the design of magnetic transmitters are optimal efficiency and minimal instantaneous power. This work proposes a systematic design approach for magnetic induction communication transmitters that operate in weakly coupled applications. The design is formulated as an optimization problem, in which the objective is minimal active and reactive power in the transmitting coil, under the constraint of a desired magnetic field at a far receiver. The free variables are the electric current densities in the transmitting coil. Considering the objective of minimal reactive power, closed form solutions are provided for the optimal current distribution. Two methods for reactive power optimization are considered. The first method computes the optimal current distribution of a flat disc coil, resulting in an analytic formula for the optimal current density as function of radius. In the second method the locations of the current loops within the transmitter coil are predefined, and the objective is to calculate the optimal amplitudes. This enables a solution for general coil geometries Eq (55). For flat coil geometries the two methods converge (Fig 5). Reactive power consumption is reduced by more than 30% in this case, compared to constant current flat coil geometries (Fig 7). The analysis is broadened to include active power losses by considering the objective of minimal apparent power. We propose a method to reduce the computational complexity of this problem by transforming it in to an equivalent problem with two free variables Eq (58), allowing a quick and efficient numeric solution. In this case the optimal current distribution enables reduction of 50% in active power, as depicted at Figs 12 and 13. Experimental results validate the theoretical results, for both reactive power and active power reduction.
